# Supplementary material for: Coccolith arrangement follows Eulerian mathematics in the coccolithophore Emiliania huxleyi
Source: PeerJ. 2018 Apr 9;6:e4608. doi: 10.7717/peerj.4608 (PMC5896503; doi:10.7717/peerj.4608)
Supplement: Supplemental Information 2 — The face numbers are a list of consecutive positive integers. The isomer number of polyhedral coccosphere was calculated using the software CaGe. The numbers in red color indicate where no polyhedra exist with predicted polygon compositions. [file peerj-06-4608-s002.docx]

**Supplementary Material**
**Supplementary Table 1** The polygon compositions of polyhedra (6-22 faces) were predicted based on Euler’s formula, and further examined using the software CaGe to obtain isomer numbers. The face numbers are a list of consecutive positive integers. The isomer number of polyhedral coccosphere was calculated using the software CaGe. The numbers in red color indicate where no polyhedra exist with predicted polygon compositions.

| face number | polygon compositions | | | isomer number |
| --- | --- | --- | --- | --- |
|  | Quadrilateral | Pentagon | Hexagon |  |
| 6 | 6 | 0 | 0 | 1 |
| 7 | 6 | 0 | 1 | 0 |
|  | 5 | 2 | 0 | 1 |
| 8 | 6 | 0 | 2 | 1 |
|  | 5 | 2 | 1 | 0 |
|  | 4 | 4 | 0 | 1 |
| 9 | 6 | 0 | 3 | 1 |
|  | 5 | 2 | 2 | 1 |
|  | 4 | 4 | 1 | 1 |
|  | 3 | 6 | 0 | 1 |
| 10 | 6 | 0 | 4 | 1 |
|  | 5 | 2 | 3 | 1 |
|  | 4 | 4 | 2 | 3 |
|  | 3 | 6 | 1 | 1 |
|  | 2 | 8 | 0 | 1 |
| 11 | 6 | 0 | 5 | 1 |
|  | 5 | 2 | 4 | 2 |
|  | 4 | 4 | 3 | 4 |
|  | 3 | 6 | 2 | 2 |
|  | 2 | 8 | 1 | 1 |
|  | 1 | 10 | 0 | 0 |
| 12 | 6 | 0 | 6 | 3 |
|  | 5 | 2 | 5 | 2 |
|  | 4 | 4 | 4 | 10 |
|  | 3 | 6 | 3 | 5 |
|  | 2 | 8 | 2 | 2 |
|  | 1 | 10 | 1 | 0 |
|  | 0 | 12 | 0 | 1 |
| 13 | 6 | 0 | 7 | 1 |
|  | 5 | 2 | 6 | 5 |
|  | 4 | 4 | 5 | 11 |
|  | 3 | 6 | 4 | 11 |
|  | 2 | 8 | 3 | 3 |
|  | 1 | 10 | 2 | 1 |
|  | 0 | 12 | 1 | 0 |
| 14 | 6 | 0 | 8 | 3 |
|  | 5 | 2 | 7 | 4 |
|  | 4 | 4 | 6 | 25 |
|  | 3 | 6 | 5 | 14 |
|  | 2 | 8 | 4 | 11 |
|  | 1 | 10 | 3 | 1 |
|  | 0 | 12 | 2 | 1 |
| 15 | 6 | 0 | 9 | 3 |
|  | 5 | 2 | 8 | 10 |
|  | 4 | 4 | 7 | 29 |
|  | 3 | 6 | 6 | 34 |
|  | 2 | 8 | 5 | 13 |
|  | 1 | 10 | 4 | 3 |
|  | 0 | 12 | 3 | 1 |
| 16 | 6 | 0 | 10 | 3 |
|  | 5 | 2 | 9 | 8 |
|  | 4 | 4 | 8 | 55 |
|  | 3 | 6 | 7 | 50 |
|  | 2 | 8 | 6 | 30 |
|  | 1 | 10 | 5 | 5 |
|  | 0 | 12 | 4 | 2 |
| 17 | 6 | 0 | 11 | 2 |
|  | 5 | 2 | 10 | 16 |
|  | 4 | 4 | 9 | 71 |
|  | 3 | 6 | 8 | 78 |
|  | 2 | 8 | 7 | 50 |
|  | 1 | 10 | 6 | 10 |
|  | 0 | 12 | 5 | 3 |
| 18 | 6 | 0 | 12 | 8 |
|  | 5 | 2 | 11 | 21 |
|  | 4 | 4 | 10 | 102 |
|  | 3 | 6 | 9 | 126 |
|  | 2 | 8 | 8 | 91 |
|  | 1 | 10 | 7 | 20 |
|  | 0 | 12 | 6 | 6 |
| 19 | 6 | 0 | 13 | 3 |
|  | 5 | 2 | 12 | 25 |
|  | 4 | 4 | 11 | 129 |
|  | 3 | 6 | 10 | 203 |
|  | 2 | 8 | 9 | 133 |
|  | 1 | 10 | 8 | 37 |
|  | 0 | 12 | 7 | 6 |
| 20 | 6 | 0 | 14 | 7 |
|  | 5 | 2 | 13 | 27 |
|  | 4 | 4 | 12 | 198 |
|  | 3 | 6 | 11 | 280 |
|  | 2 | 8 | 10 | 236 |
|  | 1 | 10 | 9 | 57 |
|  | 0 | 12 | 8 | 15 |
| 21 | 6 | 0 | 15 | 7 |
|  | 5 | 2 | 14 | 45 |
|  | 4 | 4 | 13 | 225 |
|  | 3 | 6 | 12 | 438 |
|  | 2 | 8 | 11 | 334 |
|  | 1 | 10 | 10 | 109 |
|  | 0 | 12 | 9 | 17 |
| 22 | 6 | 0 | 16 | 7 |
|  | 5 | 2 | 15 | 38 |
|  | 4 | 4 | 14 | 342 |
|  | 3 | 6 | 13 | 601 |
|  | 2 | 8 | 12 | 544 |
|  | 1 | 10 | 11 | 163 |
|  | 0 | 12 | 10 | 40 |
